# Case of Double Amputation

**Published:** 1847-06

**Authors:** 


*Case of Double Amputation.* By M. Brouzet.—M. Brouzet relates the case of a man on whom he practised amputation of both legs below the knee, the one immediately after the other, on account of injuries inflicted by a wheel of a railway carriage. The patient suffered comparatively little from the double shook of the injury and the amputations, and made a speedy and perfect recovery.—*Revue Medico-Chirurgicale de Paris.*

It has been laid down as a precept by two French surgical writers, M. Velpeau, and M. Vidal de Cassis, that the system being unable to stand the shock of a double amputation, two limbs should never be amputated at the same time, at least in such cases of injury as necessitate amputation above the wrist or ankle joints. It appears to us that there are cases, in which no general rule can be laid down, and in which the surgeon must be guided entirely by his own discretion. The propriety of operative interference must depend on the features of each individual case. Two other cases are mentioned by M. Brouzet, of recovery from simultaneous double amputations. In the Hotel des Invalides at Paris, many veterans may be seen enjoying good health, who have lost more than one limb in action, and many cases might be cited, where recovery has followed this severe mutilation.

We have heard of both limbs being amputated at the same moment, by different surgeons, in cases of this kind, but we are doubtful whether the shock to the system is less than when the two limbs are amputated successively.—*Monthly Journ. of Med. Science.*

